# Microbiome features in bronchoalveolar lavage fluid of patients with idiopathic inflammatory myopathy-related interstitial lung disease

**DOI:** 10.3389/fmed.2024.1338947

**Published:** 2024-04-03

**Authors:** Liyan Zhang, Xueqing Liu, Bijun Fan, Jiajun Chen, Jie Chen, Qiuhong Li, Xueling Wu

**Affiliations:** ^1^Department of Respiratory and Critical Care Medicine, Ren Ji Hospital, Shanghai Jiao Tong University School of Medicine, Shanghai, China; ^2^Department of Rheumatology Medicine, Ren Ji Hospital, Shanghai Jiao Tong University School of Medicine, Shanghai, China; ^3^Department of Respiratory and Critical Care Medicine, Shanghai Pulmonary Hospital, School of Medicine, Tongji University, Shanghai, China

**Keywords:** idiopathic inflammatory myopathy, interstitial lung disease, lower respiratory tract, microbiome, metagenomic next-generation sequencing

## Abstract

**Background:**

Interstitial lung disease (ILD) is a common complication of idiopathic inflammatory myopathy (IIM), which is one of the connective tissue diseases (CTD). It can lead to poor prognosis and increased mortality. However, the distribution and role of the lower respiratory tract (LRT) microbiome in patients with IIM-ILD remains unclear. This study aimed to investigate the microbial diversity and community differences in bronchoalveolar lavage fluid (BALF) in patients with IIM-ILD.

**Methods:**

From 28 June 2021 to 26 December 2023, 51 individual BALF samples were enrolled, consisting of 20 patients with IIM-ILD, 16 patients with other CTD-ILD (including 8 patients with SLE and 8 with RA) and 15 patients with CAP. The structure and function of microbiota in BALF were identified by metagenomic next-generation sequencing (mNGS).

**Results:**

The community evenness of LRT microbiota within the IIM-ILD group was marginally lower compared to the other CTD-ILD and CAP groups. Nonetheless, there were no noticeable differences. The species community structure was similar among the three groups, based on the Bray-Curtis distance between the samples. At the level of genus, the IIM-ILD group displayed a considerably higher abundance of Pseudomonas and Corynebacterium in comparison to the CAP group (*p* < 0.01, *p* < 0.05). At the species level, we found that the relative abundance of *Pseudomonas aeruginosa* increased significantly in the IIM-ILD group compared to the CAP group (*p* < 0.05). Additionally, the relative abundance of *Prevotella pallens* was significantly higher in other CTD-ILD groups compared to that in the IIM-ILD group (*p* < 0.05). Of all the clinical indicators examined in the correlation analysis, ferritin level demonstrated the strongest association with LRT flora, followed by Serum interleukin-6 level (*p* < 0.05).

**Conclusion:**

Our research has identified particular LRT microorganisms that were found to be altered in the IIM-ILD group and were significantly associated with immune function and inflammatory markers in patients. The lower respiratory tract microbiota has potential in the diagnosis and treatment of IIM-ILD.

## Introduction

Connective tissue disease-associated interstitial lung disease (CTD-ILD) and community-acquired pneumonia (CAP) are heterogeneous lung diseases (1). They are all characterized by inflammatory lesions in the lungs. Notably, CTD-ILD and CAP present significant pathogenesis and prognosis differences. Idiopathic inflammatory myopathies (IIM), a type of CTD, are a group of heterogeneous diseases that are characterized by muscle involvement and chronic inflammation of the proximal extremities. Currently, the IIM subtypes are dermatomyositis (DM), anti-synthetase syndrome (ASS), immune-mediated necrotizing myopathy (IMNM) and polymyositis (PM). DM and ASS are the two more common clinical subtypes (2). IIMs are considered to be associated with higher mortality, with an estimated ten-year survival rate of 50–90% (3). Interstitial lung disease (ILD) is a common complication of IIM and is associated with a poorer prognosis and increased mortality. However, the pathogenesis of ILD in patients with IIM is not fully understood.

For a long time, the lung was believed to be a sterile environment (4). However, with advancements in sequencing technology, this perspective has been reversed (5). Several studies have shown that a variety of microorganisms reside in the upper and lower respiratory tracts (LRTs), and the composition of these microbial communities can be altered in different respiratory disease states (6). The LRT micro-environment can be associated with lung disease including ILDs (7, 8). Previous studies have shown that the lung microbiome of patients with idiopathic pulmonary fibrosis (IPF), a type of ILD, differs from that of healthy individuals (9). IPF patients had a higher abundance of Haemophilus, Streptococcus, Neisseria and Veillonella compared to healthy individuals (10). In a cohort study, researchers sequenced the lung microbiota in IPF patients’ bronchoalveolar lavage fluid (BALF) samples and reported the presence of Streptococcus and Staphylococcus. It was associated with faster progression of the disease when these two species exceeded a certain threshold (11). However, the role of the LRT microbiome in patients with IIM-ILD is still unclear.

In addition to the role of the microbiome in the development of lung disease, several inflammatory and immune have been shown to affect disease progression in ILD. Levels of certain serum markers such as ferritin, IL-8, IL-10, IL-18, and TNF-α, are correlated with disease activity in ILD (12). Therefore, this study aimed to compare the microbial diversity and community differences in the lower respiratory microbiome of patients with IIM-ILD, other CTD-ILD and CAP. We also included a large number of inflammation and immune-related markers for correlation analysis. The goal is to identify potential biomarkers that may influence the development of IIM-ILD and to provide direction and a basis for early diagnosis and treatment of the disease.

## Materials and methods

### Ethics statement and subjects

The study was approved by the Ethics Committee of Ren Ji Hospital, Shanghai Jiao Tong University School of Medicine, Shanghai, China and Shanghai Pulmonary Hospital, School of Medicine, Tongji University, Shanghai, China. A total of 51 adult subjects were enrolled from 28 June 2021 to 26 December 2023 in the Department of Respiratory, Ren Ji Hospital and Shanghai Pulmonary Hospital once diagnosed after multidisciplinary consultation which was comprised of 20 patients with IIM-ILD, 16 patients with other CTD-ILD, and 15 patients with CAP. Among the 16 other CTD-ILD patients, there were 8 patients with systemic lupus erythematosus (SLE) and 8 patients with rheumatoid arthritis (RA). The patient selection criteria for IIM-ILD and CTD-ILD groups were as follows. Adults aged from 18 to 85 years old who met the latest diagnostic criteria of the American College of Rheumatology and the European Federation of Rheumatological Societies and have ILD on high-resolution CT scan imaging and lung biopsy were eligible for inclusion (13–15). Patients with CAP were enrolled according to the Diagnosis and treatment of community-acquired pneumonia guidelines of the American Thoracic Society and Infectious Diseases Society of America (16). All subjects were newly diagnosed patients who had not been treated with antibiotics, hormones, immunosuppressants and other drugs before being obtained alveolar lavage fluid. All subjects signed informed consent. The medical history of all participants was collected and routine preoperative examinations including physical examination, lung CT scan, electrocardiogram, lung function examination, blood count and coagulation function analysis were performed before undergoing bronchoscopy in Ren Ji Hospital and Shanghai Pulmonary Hospital. The clinic characteristics and information are shown in Table 1. Fifty one individual BALF samples were collected and sent for mNGS examination (Dinfectome Inc., Nanjing, China).

**Table 1:** Basic characteristics and clinic parameters of the IIM-ILD, other CTD-ILD, and CAP group.

	IIM-ILD	Other CTD-ILD	CAP	*p*-value
	(*n* = 20)	(*n* = 16)	(*n* = 15)	
Age	57.05 (13.94)	60.19 (11.52)	57 (13.24)	0.75
Gender, n, (% male)	9 (45)	6 (37.5)	10 (66.7)	0.24
Smokers, n (%)	4 (20)	6 (37.5)	6 (40)	0.37
*Comorbidities*				
Hypertension, n (%)	6 (30)	6 (37.5)	5 (33.3)	0.89
Diabetes, n (%)	3 (15)	6 (37.5)	6 (40)	0.19
Coronary heart disease, n (%)	3 (15)	1 (6.25)	4 (26.7)	0.29
Laboratory findings				
*Peripheral blood*				
WBC (×109)	8.36 (2.06)	6.77 (1.38)	7.09 (2.55)	0.05
Neutrophiles(×109)	6.53 (2.19)	4.67 (1.68)	4.99 (2.45)	<0.05
Lymphocytes(×109)	1.31 (1.01)	1.54 (0.71)	1.48 (0.70)	0.37
*BALF cell count*				
Neutrophile percentages(%)	29.95 (29.16)	46.56 (28.54)	23.67 (31.77)	<0.05
Lymphocyte percentages(%)	9.15 (17.18)	5.38 (7.17)	8.07 (19.11)	0.30
Macrophage percentages(%)	60.50 (28.84)	48.00 (26.25)	66.67 (32.40)	0.13
*Inflammatory makers*				
CRP (ug/L)	14.37 (16.24)	10.15 (11.93)	17.43 (26.17)	0.43
PCT (mg/L)	0.10 (0.17)	0.06 (0.08)	0.05 (0.03)	0.95
*Peripheral blood cytokine*				
IL2	2.66 (3.35)	1.62 (0.90)	6.08 (16.54)	0.59
IL4	1.97 (1.50)	1.58 (0.59)	5.60 (10.72)	0.50
IL6	26.97 (36.74)	16.05 (33.08)	11.09 (12.59)	0.70
IL8	54.52 (71.63)	32.84 (52.52)	42.06 (90.92)	0.58
IL10	9.05 (9.13)	3.59 (2.04)	4.28 (6.68)	0.09
TNF-α	6.26 (18.41)	1.49 (0.95)	7.14 (17.36)	0.13
IFN-α	2.70 (3.83)	1.50 (0.78)	4.81 (11.15)	0.95
INF-γ	1.91 (0.85)	1.89 (1.16)	1.96 (0.65)	0.78
KL6	2,153 (2709)	1033.87 (495.40)	252.40 (272.07)	<0.001
Ferritin	257.58 (318.28)	288.99 (352.54)	177.71 (121.97)	0.83
*Serum immunoglobulin*				
IgA	2.21 (0.91)	2.57 (0.99)	2.63 (1.03)	0.38
IgM	1.29 (0.74)	1.18 (0.59)	1.23 (0.67)	0.99
IgG	13.20 (3.74)	13.17 (2.79)	13.88 (2.47)	0.86
IgE	50.16 (51.65)	48.62 (41.96)	103.14 (179.38)	0.84

Age, blood white cell count, neutrophils, lymphocytes, BALF neutrophils, lymphocytes, macrophages, CRP, PCT, IL-2, IL-4, IL-6, IL-8, TNF-α, IFN-α, INF-γ, KL-6, ferritin, IgA, IgM, IgG and IgE are expressed as the mean (SD). Between-group comparisons were made with the chi-square test (χ2 Test) for normally distributed continuous variables and with the Kruskal–Wallis (KW) test for non-normally distributed continuous variables. The font for parameters with *p* < 0.05 has been bolded.

### Specimen collection

BALF was obtained from 51 participants. Samples were collected from patients according to standard procedures (17). After local lidocaine anesthesia of the patient’s throat, the fiberoptic bronchoscope was introduced. The lung was lavaged with room temperature sterile saline several times through the fiberoptic bronchoscope, 20 mL each time, a total of 100 mL. The BALF recovery rate was more than 40%. Lavage fluid was retrieved by negative pressure suction, and 5 mL of the sample was removed from the recovered solution into a sterile nucleic acid-free DNA sampling tube and stored immediately at −80°C.

### Nucleic acid extraction

BALF samples were collected from patients according to standard procedures. The TIANamp Magnetic DNA Kit (Tiangen) was used to extract DNA. The quantity and quality of DNA were, respectively, assessed using the Qubit (Thermo Fisher Scientific) and NanoDrop (Thermo Fisher Scientific).

### Library preparation and sequencing

DNA libraries were prepared using the Hieff NGS C130P2 OnePot II DNA Library Prep Kit for MGI (Yeasen Biotechnology) according to the manufacturer’s protocols. Agilent 2,100 was used for quality control and DNA libraries were 50 bp single-end sequenced on MGISEQ-200.

### Bioinformatics analysis

Raw sequencing data was split by bcl2fastq2 (version 2.20), and high-quality sequencing data were generated using Trimmomatic (version 0.36) by removing low-quality reads, adapter contamination, duplicated and shot (length; 36 bp) reads. Human host sequences were subtracted by mapping to the human reference genome (hs37d5) using bowtie2 (version 2.2.6). Reads that could not be mapped to the human genome were retained and aligned with the microorganism genome database for microbial identification by Kraken (version 2.0.7), and species abundance estimating by Bracken (version 2.5.0). The microorganism genome database contained genomes or scaffolds of bacteria, fungi, viruses and parasites (download from GenBank release 238, ftp://ftp.ncbi.nlm.nih.gov/genomes/genbank/).

### Statistical analysis

Statistical analysis was performed by R software (version 4.0.1). Comparisons of basic characteristics and clinic parameters in different groups were made with the chi-square test (χ2 Test) for normally distributed continuous variables and with the Kruskal–Wallis (KW) test for non-normally distributed continuous variables. Shannon index based on the taxonomic profile of each sample was used to assess αdiversity and the Bray-Curtis method was used to assess β diversity. Wilcoxon rank-sum test was used to compare between IIM-ILD, other CTD-ILD and CAP patients, and the principal coordinate analysis (PCoA) plot was subsequently visualized. PREMANOVA was performed by the R package “vegan” to analyze Bray-Curtis distance in different IIM-ILD, other CTD-ILD and CAP patient groups. The Kruskal-Wallis rank sum test was used to test the relative abundance difference of each group at the genus level. Genera with average relative abundances greater than 1% and penetrance greater than 40% among all samples were compared. Spearman’s correlations between clinical features and the relative genus abundances were calculated by the R package “cor.test,” and FDR correction was adopted to adjust all *p* values. Linear discriminant analysis of effect size (LEfSe) was used to evaluate the statistical differences in the relative abundance of microorganisms among the groups.

## Results

### Participants characteristics

This study included 20 patients with IIM-ILD, 16 patients with other CTD-ILD and 15 patients with CAP. Serological analyses of each patient, including blood routine examination, C-reactive protein (CRP), Procalcitonin (PCT), ferritin, Krebs Von den Lungen-6 (KL-6), serum interleukin (IL) and immunoglobulin (Ig) were performed using standard methods. Meanwhile, the alveolar lavage fluid of LRT was obtained by bronchoscopy and cell classification was performed. The clinical characteristics and information are shown in attached Table 1. There was a significant difference in peripheral neutrophil count and BALF neutrophil percentage (*p* < 0.05), as well as a highly significant difference in KL-6 levels (*p* < 0.001) among the three groups. No significant differences were observed in any other clinical indicators.

### The composition of LRT microbiota in IIM-ILD, other CTD-ILD and CAP groups

**Figure 1:**
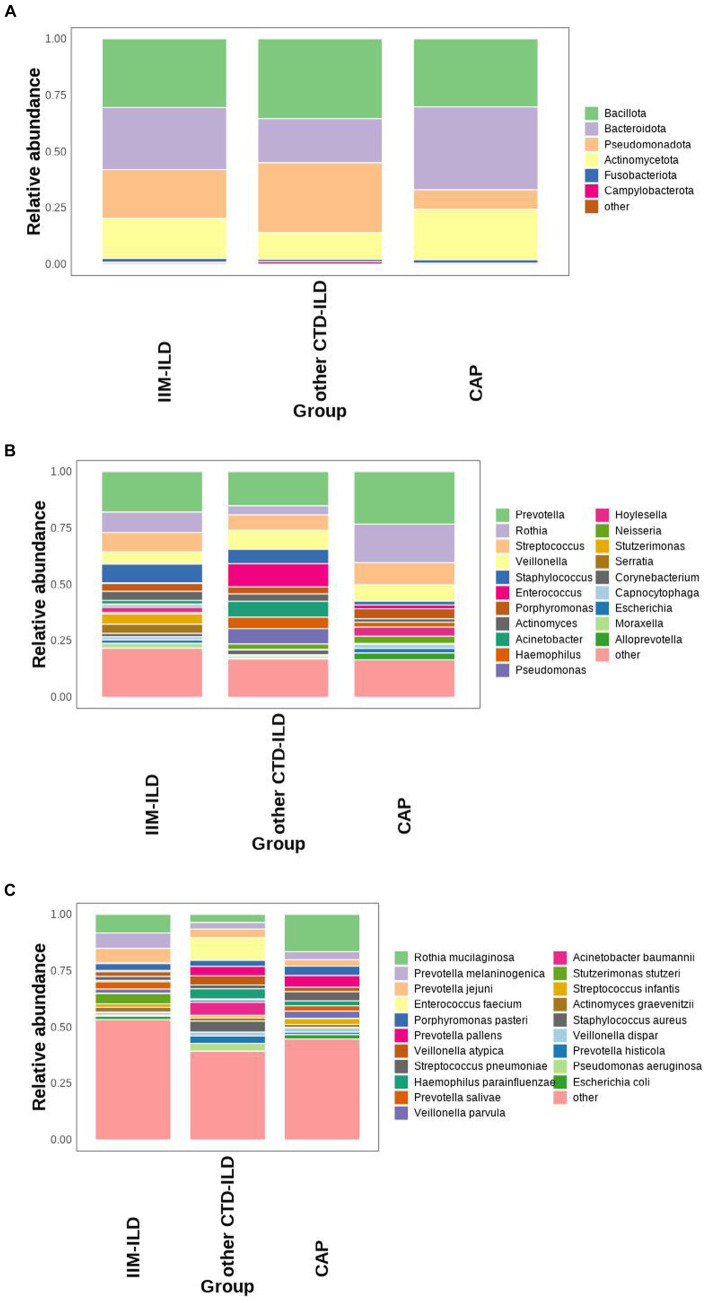
Microbial composition at phylum, genus, and species level in IIM-ILD, other CTD-ILD, and CAP groups. **(A)** Stacked bar charts showing the relative abundance of the major components in the three groups at the phylum level. **(B)** Stacked bar charts showing the relative abundance of the major components in three groups at the genus level. **(C)** Box map of multispecies differences tests showing the relative abundance of the top 20 species at the species level.

We first analyzed the composition of bacterial compositions of the patients with IIM-ILD, other CTD-ILD and CAP. The most abundant phyla were Bacillota, Bacteroidota, Pseudomonadota and Actinomycetota. The top 20 microbiomes in each of the three groups were listed at the genus and the species levels. In the IIM-ILD group, Prevotella occupied an overwhelming predominance with a relative abundance of 17.94%, followed by Rothia (8.99%), Streptococcus (8.64%), Staphylococcus (8.30%), and Veillonella (5.42%). In the other CTD-ILD group, the relative abundance of Prevotella also was the highest (15.16%), but the next was Enterococcus (10.31%), followed by Veillonella (8.46%), Acinetobacter (7.03%) and Streptococcus (6.82%). In the CAP patients, the top three microbiomes were Prevotella (23.28%), Rothia (17.09%) and Streptococcus (9.85%). The top five species in relative abundance in the IIM-ILD group were *Rothia mucilaginosa* (8.24%), *Prevotella melaninogenica* (6.85%), Prevotella jejuni (6.48%), Stutzerimonas stutzeri (4.55%), and *Prevotella salivae* (3.24%). In contrast, in the CTD-ILD group, these were *Enterococcus faecium* (10.14%), *Acinetobacter baumannii* (5.80%), *Staphylococcus aureus* (4.93%), *Haemophilus parainfluenzae* (4.55%), and *Prevotella pallens* (4.18%). The most common species in the CAP group were *Rothia mucilaginosa* (16.54%), *Prevotella pallens* (5.15%), Porphyromonas pasteri (4.19%), *Streptococcus pneumoniae* (4.08%), and *Prevotella melaninogenica* (3.60%). These findings are illustrated in Figure 1.

### The diversity of LRT microbiota in three groups

**Figure 2:**
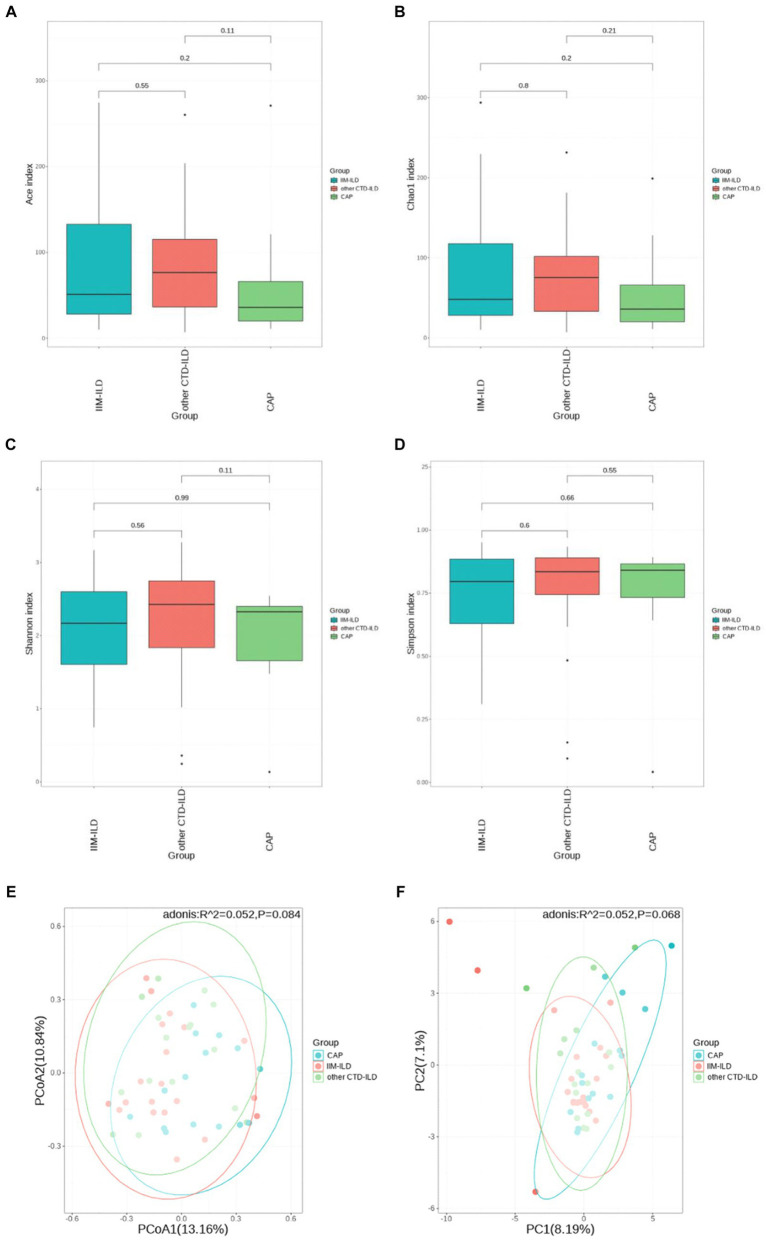
Diversity of LRT microbial flora among IIM-ILD, other CTD-ILD, and CAP groups. **(D)** The α diversity of BALF microbial flora in samples collected from three groups. **(A)** Ace index. **(B)** ChaO1 index. **(C)** Shannon index. **(D)** Simpson index. **(F)** The β diversity of BALF microbial flora in samples collected from three groups. The PCoA plot **(E)** and PCA plot **(F)**.

Alpha diversity analysis was based on Shannon, Simpson, Chao1, and ACE indicators. The Chao1 and ACE indices which represent the community evenness of lower respiratory tract microbiota in the IIM-ILD group were lower than other CTD-ILD group and CAP group, but not significant (*p* > 0.05, Kruskal–Wallis test) (Figures 2A–D). Furthermore, the PCA and PCoA based on the Bray–Curtis distances showed that the beta diversity of the lower respiratory tract microbiota did not differ significantly between the three groups (Figures 2E,F) (*p* > 0.05, ADONIS analysis). It is suggested that the community structure of the lower respiratory flora is similar in the three diseases.

### Difference of main abundant bacterial taxa

**Figure 3:**
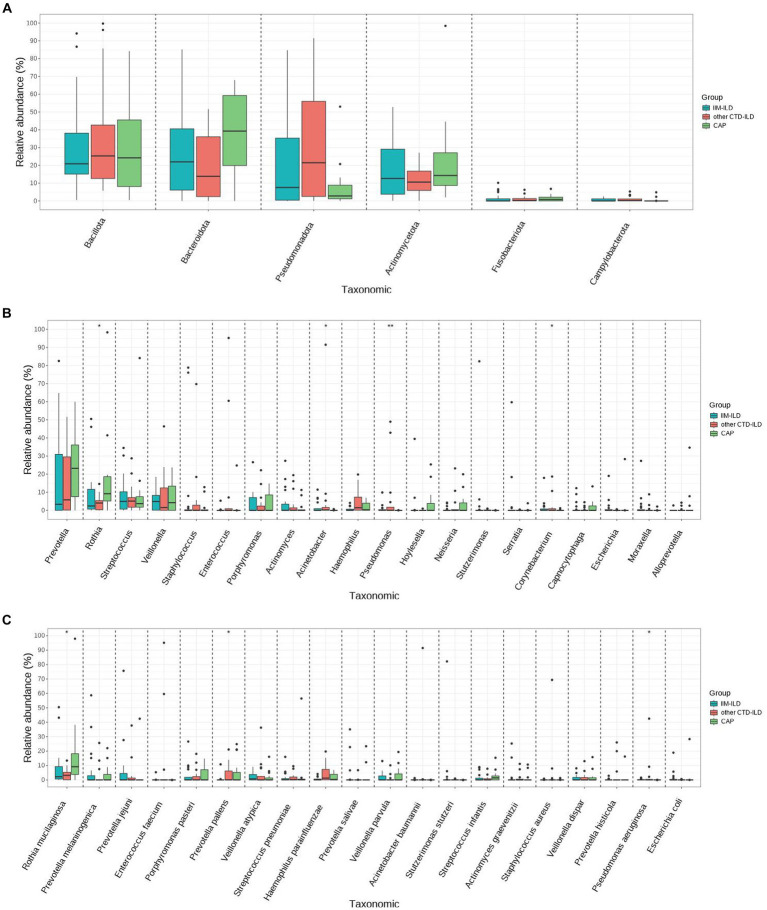
Difference of main abundant bacterial taxa among IIM-ILD, other CTD-ILD, and CAP groups. **(A)** Box map of multispecies differences tests showing the relative abundance at the phylum level.**(B)** Box map of multispecies differences tests showing the relative abundance of the top 20 bacteria at the genus level. **(C)** Box map of multispecies differences tests showing the relative abundance of the top 20 bacteria at the species level.

We did not find the statistically significant differences in LRT microbial flora at phylum level among the three groups (*p* > 0.05, Kruskal–Wallis test) (Figure 3A). At genus level, it was observed that the relative abundance of Pseudomonas and Corynebacterium in IIM-ILD group were significantly higher than that in CAP group (*p* = 0.007, *p* = 0.042, Kruskal-Wallis test). But not significantly different from the other CTD-ILD group. Furthermore, we found that the relative abundance of Rothia in the CAP group was significantly higher than that in other CTD-ILD groups (*p* = 0.039, Kruskal-Wallis test) while the relative abundance of Acinetobacter was significantly lower than that in other CTD-ILD group (*p* = 0.015, Kruskal-Wallis test). However, these two were not significantly different from the IIM-ILD group (Figure 3B).

At the species level, we found that the relative abundance of *Pseudomonas aeruginosa* increased significantly in the IIM-ILD group compared to the CAP group (*p* = 0.017, Kruskal-Wallis test), but not significantly different from the other CTD-ILD group. We also observed that the relative abundance of relative abundance of *Prevotella pallens* was significantly higher in other CTD-ILD compared to that in IIM-ILD (*p* = 0.038, Kruskal-Wallis test). Furthermore, we found that the relative abundance of *Rothia mucilaginosa* in the CAP group was significantly higher than that in other CTD-ILD group (*p* = 0.039, Kruskal-Wallis test), but was not significantly different from the IIM-ILD group (Figure 3C).

### The dominant microbial taxa in the IIM-ILD, other CTD-ILD, and CAP group

**Figure 4:**
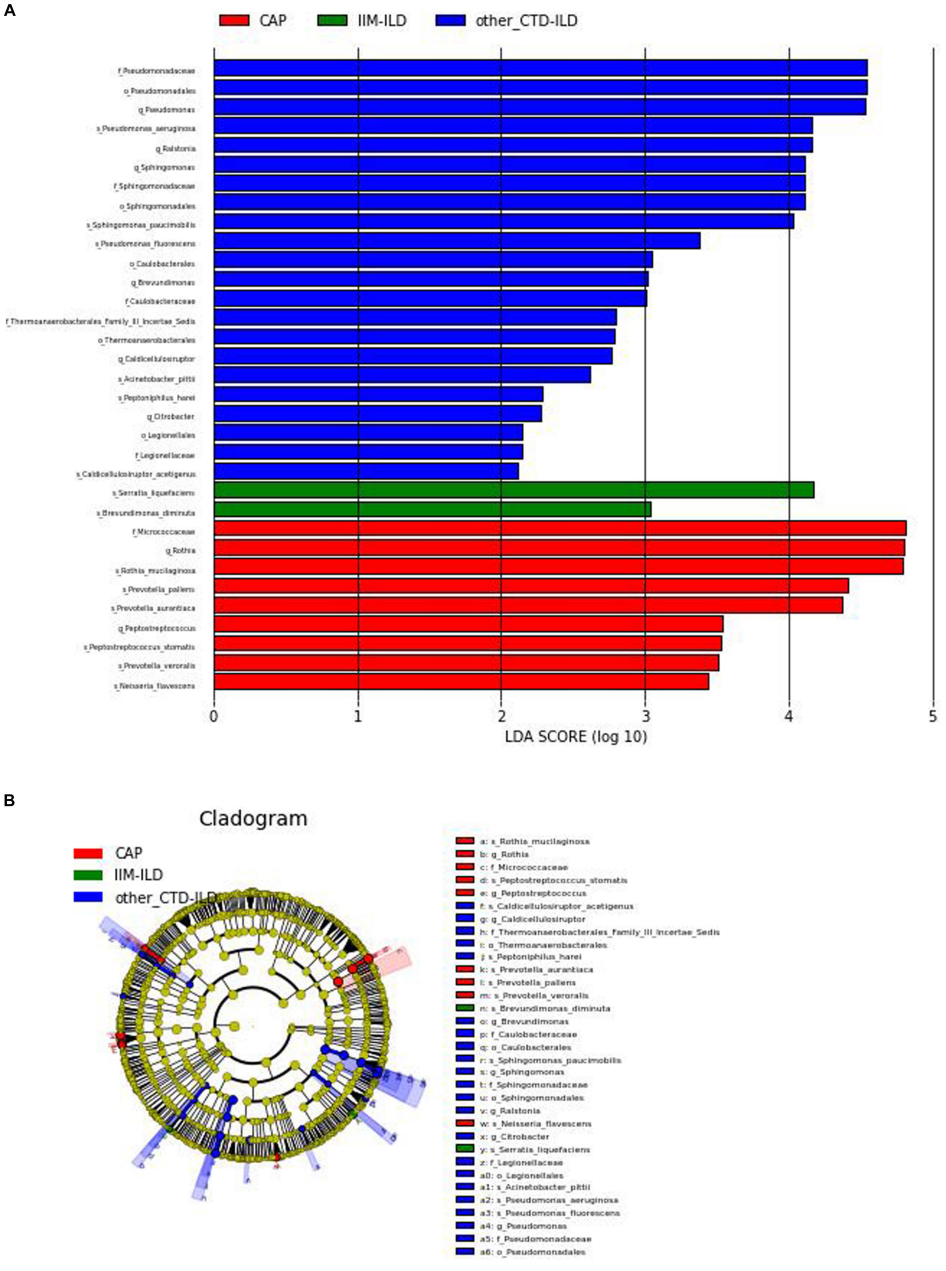
Linear discriminant analysis Effect Size (LEfSe). **(A)** LDA shows distinct lung microbiome composition associated with IIM-ILD, other CTD-ILD, and CAP. LDA scores as calculated by LEfSe of taxa differentially abundant in different disease groups. Only taxa with LDA scores of more than two and *p-*value <0.05 are shown here. **(B)** Cladogram. Circles radiating from the inside out represent taxonomic levels from phylum to genus (or species). At different taxonomy levels, each small circle represents a classification belonging to that level, and the size of the small circle diameter is proportional to the size of the relative abundance. Coloring principle: no significant differences in species uniform coloring yellow Color, different species Biomarker follows group color, red nodes represent important microbial groups in the red group, green nodes represent important microbial groups in the green group.

Thirty three discriminative features were identified by the LEfse analysis (Linear discriminant analysis Effect Size, LDA). Among them, 2 taxa were discriminative for IIM-ILD patients, 22 taxa for other CTD-ILD patients, and 9 taxa for CAP patients (Figure 4). Biomarker names, LAD scores and log values were shown in the figure.

At the genus level, Pseudomonas was the most representative bacteria in the other CTD-ILD group while Rothia was predominant in the CAP group. At the species level, there was a preponderance of *Serratia liquefaciens* (LDA score (log10) > 4) in the IIM-ILD group. And in other CTD-ILD group, *Pseudomonas aeruginosa* was predominant(LDA score (log10) > 4). The microbiome of CAP patients showed a very high abundance of *Rothia mucilaginosa*, *Prevotella pallens* and *Prevotella aurantiaca* (LDA score (log10) > 4) (Figure 4).

### The correlation between microbial taxa and environmental factor

**Figure 5:**
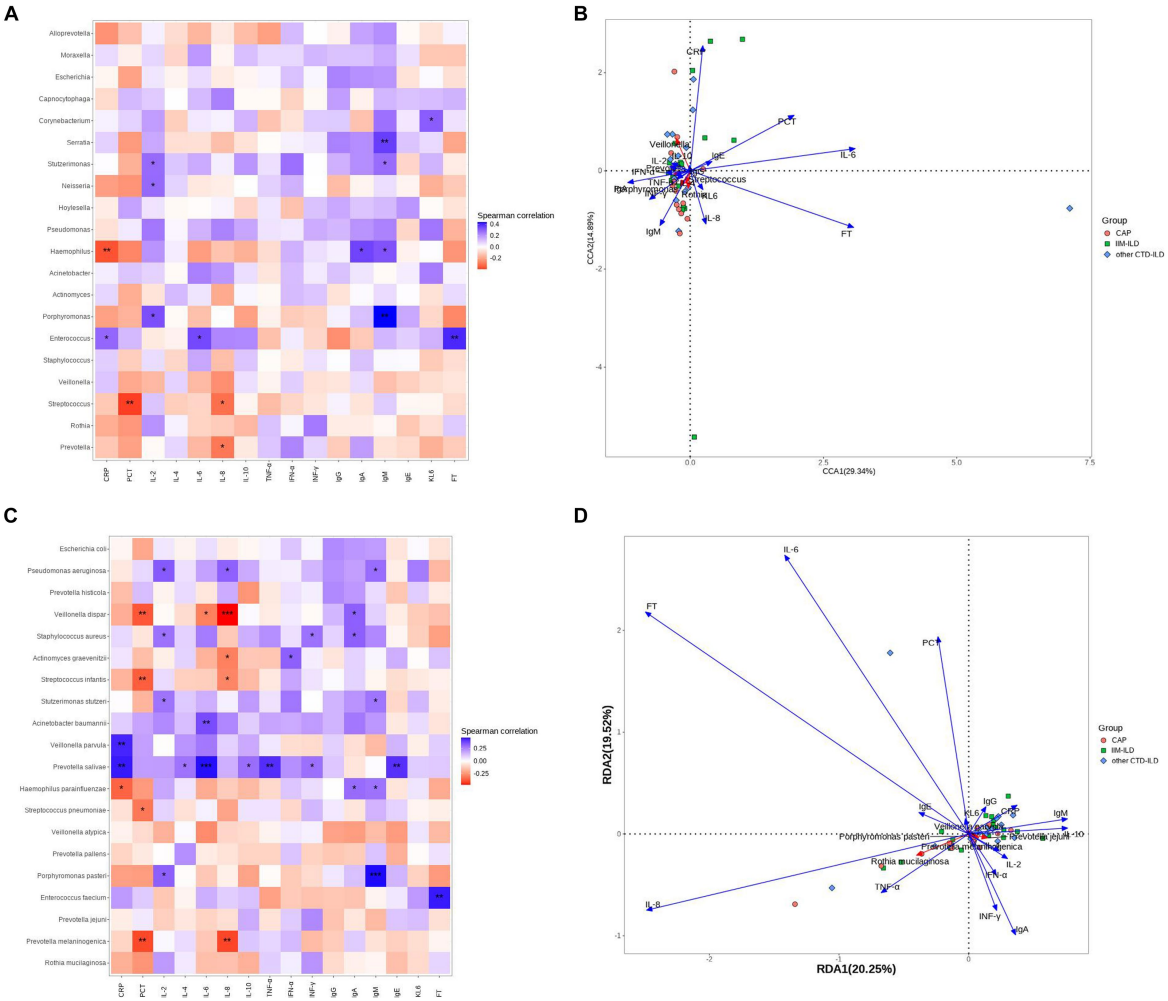
Correlation analysis between microbial taxa and clinical indicators. **(A)** Heatmap showing the top 20 abundant genera in the three groups. The horizontal coordinate indicates environmental factors, and the vertical coordinate indicates species. The correlation between the environmental factors and the species was intuitively expressed through color depth. Black stars in the blocks represent the *means *p* < 0.05, **means *p* < 0.01. **(B)** RDA analysis is mainly used to analyze the relationship between species and environmental factors. The Angle between environmental factors is a positive correlation when it is acute, and a negative correlation when it is obtuse. The longer the ray of the environmental factor, the greater the influence of the factor. **(C)** Heatmap showing the top 20 abundant species in the three groups of samples. Black stars in the blocks represent the *means *p* < 0.05, **means *p* < 0.01. **(D)** RDA analysis is mainly used to analyze the relationship between species and environmental factors.

Eventually, we use Spearman correlation analysis to further elucidate the relationship between microbial species in the LRT of all the samples and immune, inflammation-related clinical indicators. A two-dimensional sorting map was generated from the results. Ferritin levels were found to have the strongest correlation with LRT flora out of all the clinical indicators included in this analysis,followed by Serum interleukin-6 level (Figures 5B,D).

Firstly, We focused on the species and genera that differ between the three groups. At the genus level, only Corynebacterium showed a strong correlation with clinical indicators, displaying a positive correlation with KL-6 (*p* < 0.05). At species level, the relative abundance of *Pseudomonas aeruginosa* was positively correlated with IL2, IL-8 and IgM levels (p < 0.05). Furthermore, several genera and species presented significant correlations with clinical indicators aside from those differing among the three groups. Notably, the relative abundance of Porphyromonas pasteri showed a strong positive correlation with IgM levels (*p* < 0.001). Meanwhile, *Prevotella salivae* exhibited a strong positive correlation with IL-6 level(p < 0.001), and *Veillonella dispar* showed a significant positive correlation with IL-8 level (*p* < 0.001) (Figures 5A,C).

## Discussion and conclusion

Microbiomes exist in a symbiotic relationship with their human hosts, playing a crucial role in maintaining homeostasis and immune function through crosstalk with the host immune system (18). With the change in human living conditions and lifestyles, the symbiotic imbalance between human hosts and human microbiota has resulted in a sharp increase in immune-mediated diseases (19). Idiopathic inflammatory myopathies (IIMs) are a heterogeneous group of connective tissue diseases (CTDs) that exhibit chronic inflammation in the proximal muscles of the extremities. Dermatomyositis (DM) and anti-synthetase syndrome (ASS) are the two more common clinical subtypes. Interstitial lung diseases (ILDs) are the most common comorbidity of CTDs and are a set of disorders with interstitial lung involvement, characterized by histology. The clinical manifestations are dyspnea, cough, hypoxia and impaired lung function. More than half of patients with IIM develop ILD (20). However, the pathogenesis of ILDs remains unclear. Ecological imbalance is thought to be one of the important reasons (21). The microbiota has been observed to be altered in IIMs and associated with different treatments (22). Patients with IIM are extremely susceptible to co-infection due to multiple predispositions (such as immunosuppressive drugs, dysphagia, esophageal involvement and gastroesophageal reflux, etc). Interstitial lung disease and infection co-lead to increased disease severity and mortality in IIM patients.

Recently, with the rapid advances in Sequencing technology, metagenomics research using the Next Generation Sequencing technology can quickly and accurately obtain a large number of microbial gene data and rich microbial research information. Consequently, this technique has become a vital tool in investigating microbial diversity and community characteristics (23). However, limited research has been carried out on the microbial composition and diversity of the lower respiratory tract (LRT) in IIM-ILD. Few studies thoroughly compared the differences between patients with IIM-ILD, other CTD-ILD, and CAP. In the present study, samples of alveolar lavage fluid (BALF) were, respectively, obtained from the LRT of patients with IIM-ILD, other CTD-ILD, and CAP. We subsequently analyzed the composition of microbial taxa and their relationship with environmental factors using metagenomic sequencing.

In this study, Chao1 and ACE indices showed that the community evenness of LRT microbiota in the IIM-ILD group was slightly lower than that in the other CTD-ILD and CAP groups. However, there were no significant differences in alpha diversity and beta diversity among the three groups. We found that the species community structure of LRT microbiota was similar in IIM-ILD, other CTD-ILD and CAP groups. Previous research reported that patients with dermatomyositis had lower microbial diversity compared with healthy controls. Results from alpha and beta diversity showed a clear difference in the bacterial richness and community between IIM patients and healthy controls (24). However, our results were not entirely consistent with some researches. There were several possible reasons for this: (1) using alveolar lavage fluid instead of fecal samples; (2) small sample size; (3) choosing the other CTD-ILD and CAP patients as a control group, not healthy individuals.

Our study showed there were some shared microbiomes such as Prevotella, Rothia, Streptococcus, Veillonella, Staphylococcus, Acinetobacter, Pseudomonas, and Corynebacterium in the IIM-ILD, the other CTD-ILD and CAP groups. The abundances of Pseudomona and Corynebacterium in the IIM-ILD group were significantly higher than that in the CAP group, but not different from the other CTD-ILD group. Furthermore, we found that the relative abundance of Rothia in the CAP group was significantly higher than that in other CTD-ILD group while the relative abundance of Acinetobacter was significantly lower than that in other CTD-ILD group. However, these two were not significantly different from the IIM-ILD group. At the species level, the results showed that there was a significant difference in the abundance of *Prevotella pallens*, *Rothia mucilaginosa* and *Pseudomonas aeruginosa* among the three groups. We observed that the relative abundance of relative abundance of *Prevotella pallens* was significantly higher in other CTD-ILD compared to that in IIM-ILD. We found that the relative abundance of *Rothia mucilaginosa* in the CAP group was significantly higher than that in other CTD-ILD group, but was not significantly different from the IIM-ILD group. We also found that the relative abundance of *Pseudomonas aeruginosa* increased significantly in the IIM-ILD group compared to the CAP group, but not significantly different from the other CTD-ILD group. *Pseudomonas aeruginosa* was nearly depleted in the CAP group.

Prevotella is a common gram-negative obligate anaerobic bacterium that was first described by Shar and Collins in 1990. It is found in several parts of the human body, including the respiratory tract (25). Several members of Prevotella have been implicated in a variety of diseases, including infections and inflammatory autoimmune diseases (26). A previous study reported a significant difference in the decreased mean relative proportion of Prevotella in COPD and ILD patients compared to lung cancer patients (27). Our results showed that patients with CAP had higher prevotella abundance than those patients with CTD-ILD including IIM-ILD, although there was no significant difference. Prevotella was negatively correlated with serum levels of IL8. Anujit Sarkar reported that Prevotella was negatively associated with IL8 (28). Our result was consistent. Moreover, we observed that *Prevotella Salivae* had a strong positive correlation with CRP and IL6. No association was found between *Prevotella pallens* and any clinical indicators. Prevotella is known to promote the production of inflammatory cytokines, but the genus contains many different species, some of which may play different roles than other members. Yukun He reported that the abundance of Prevotella was decreased in both CAP and CTD-ILD patients and was negatively correlated with white blood cell counts and CRP levels (1). Unfortunately, we did not get that result. Interestingly, in a mouse co-infection model, airway Prevotella was found to promote toll-like receptor-dependent neutrophils activation and accelerate the clearance of *Streptococcus pneumoniae* from the lungs (29).

*Rothia mucilaginosa*, a gram-positive bacterium belonging to the Micrococcaceae family, normally distributes in the human oral cavity and upper respiratory tract. It is an opportunistic pathogen. Although this organism is considered to be low virulent, it causes disease in immunocompromised hosts (30). In a cohort study of adults with bronchiectasis, researchers found that the abundance of Rothella was inversely associated with some pro-inflammatory markers in serum. *Rothia mucilaginosa* inhibits the activation of NF-κB pathway by reducing the phosphorylation of IκBα, thereby inhibiting the expression of NF-κB target genes. It indicated that *Rothia mucilaginosa* could inhibit the proinflammatory response (31). In our study, the abundance of *Rothia mucilaginosa* was deceased in the other CTD-ILD group compared to the CAP group. However, we did not find any correlation between *Rothia mucilaginosa* and pro-inflammatory factors.

In a single-center study, *Pseudomonas aeruginosa* was found to be the most common bacteria in the airways of patients with non-cystic fibrosis bronchiectasis. PPI use and longer duration of bronchiectasis were confirmed to be independent risk factors. The presence of *Pseudomonas aeruginosa* infection or colonization is often associated with whether the patient receives immunosuppressive therapy. It is also easy to find colonized bacteria in the airway of immunocompromised people (32, 33). In our study, we found the relative abundance of *Pseudomonas aeruginosa* increased in IIM-ILD patients. It had a positive association with IL2, IL8, and IgM. Alberto Ricci observed that lung colonization of *Pseudomonas aeruginosa* was present in nearly one-third of CTD-ILD patients (34). These patients showed significant lung involvement and severe impairment of lung function. It suggested the possible relationship between the *Pseudomonas aeruginosa* colonization and the progression and severity of CTD-ILD.

Interestingly, after ruling out infection, we observed elevated peripheral neutrophils in some patients with IIM-ILD, albeit at a normal level. There was an increased BALF neutrophil percentage in IIM-ILD and other CTD-ILD patients. We speculated that it may be associated with worsened disease and a more fibrotic profile in CTD-ILD (35, 36).

In addition, as the 16 CTD-ILD included patients are 8 with SLE and 8 with RA, we further analyzed the differences between the microbiomes of these two subgroups. The results showed no significant difference in alpha and beta diversity between RA and SLE subgroups. Furthermore, we found that the relative abundance of Veillonella in the RA subgroup was significantly higher than that in the SLE subgroup at the genus level (*p* < 0.05, Kruskal-Wallis test) while the relative abundance of *Staphylococcus aureus* was significantly lower than that in SLE subgroup (p < 0.05, Kruskal-Wallis test). The relative abundance of *Staphylococcus aureus* was positively correlated with IL2 and INF-γ levels (*p* < 0.05). Due to the limited sample size, these results are worth exploring, so in future studies, we will further expand the sample size in this direction to get a more reliable conclusion.

In our study, the distribution and pathogenesis of the microbiome in LRT of patients with IIM-ILD were not deep enough, although we investigated the composition of the flora in the lower respiratory tract of IIM-ILD and identified some species that differ from other groups. Additionally, we found some clinical indicators that are of diagnostic value. To confirm the abundance and mechanism of certain microbiomes in the LRT of patients with IIM-ILD, it is necessary to expand the sample size and further CTD subgroup analysis. We will conduct a large prospective cohort study combined with clinical data.

## Data availability statement

The original contributions presented in the study are included in the article/supplementary material, further inquiries can be directed to the corresponding author.

## Ethics statement

The studies involving humans were approved by Ethics Committee of Ren Ji Hospital, Shanghai Jiao Tong University School of Medicine. The studies were conducted in accordance with the local legislation and institutional requirements. The participants provided their written informed consent to participate in this study.

## Author contributions

LZ: Data curation, Formal analysis, Writing – original draft. XL: Investigation, Methodology, Writing – review & editing. BF: Investigation, Methodology, Writing – review & editing. JiaC: Investigation, Methodology, Writing – review & editing. JieC: Methodology, Investigation, Writing – review & editing. QL: Data curation, Investigation, Methodology, Project administration, Supervision, Writing – original draft. XW: Formal analysis, Funding acquisition, Project administration, Writing – original draft.
